# Supplementary material for: Immune suppressive activity of myeloid-derived suppressor cells in cancer requires inactivation of the type I interferon pathway
Source: Nat Commun. 2021 Mar 19;12:1717. doi: 10.1038/s41467-021-22033-2 (PMC7979850; doi:10.1038/s41467-021-22033-2)
Supplement: Supplementary file 1 — Supplementary Information [file 41467_2021_22033_MOESM1_ESM.pdf]

Supplementary information

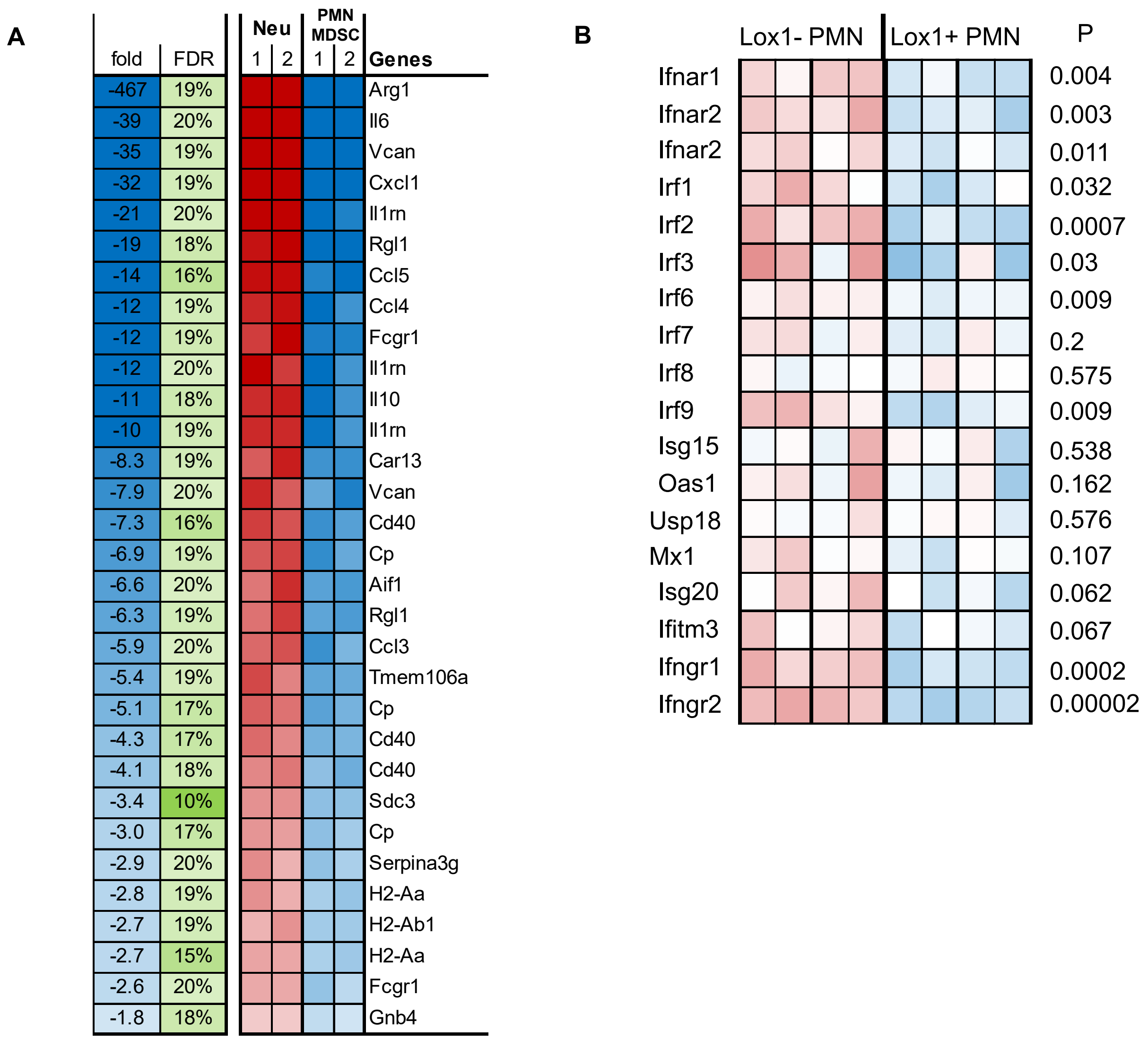

Supplementary Figure 1. List of genes involved in IFN1 signaling in PMN-MDSCs versus PMNs. (A) Affymetrix microarray analysis of genes involved in type 1 interferon signaling pathway in mouse PMN-MDSCs and neutrophils. False discovery rate (FDR) was calculated in Fisher exact test. (B) RNAseq analysis of genes involved in type 1 interferon signaling pathway in human LOX1+ PMN-MDSC and LOX1 − PMN.

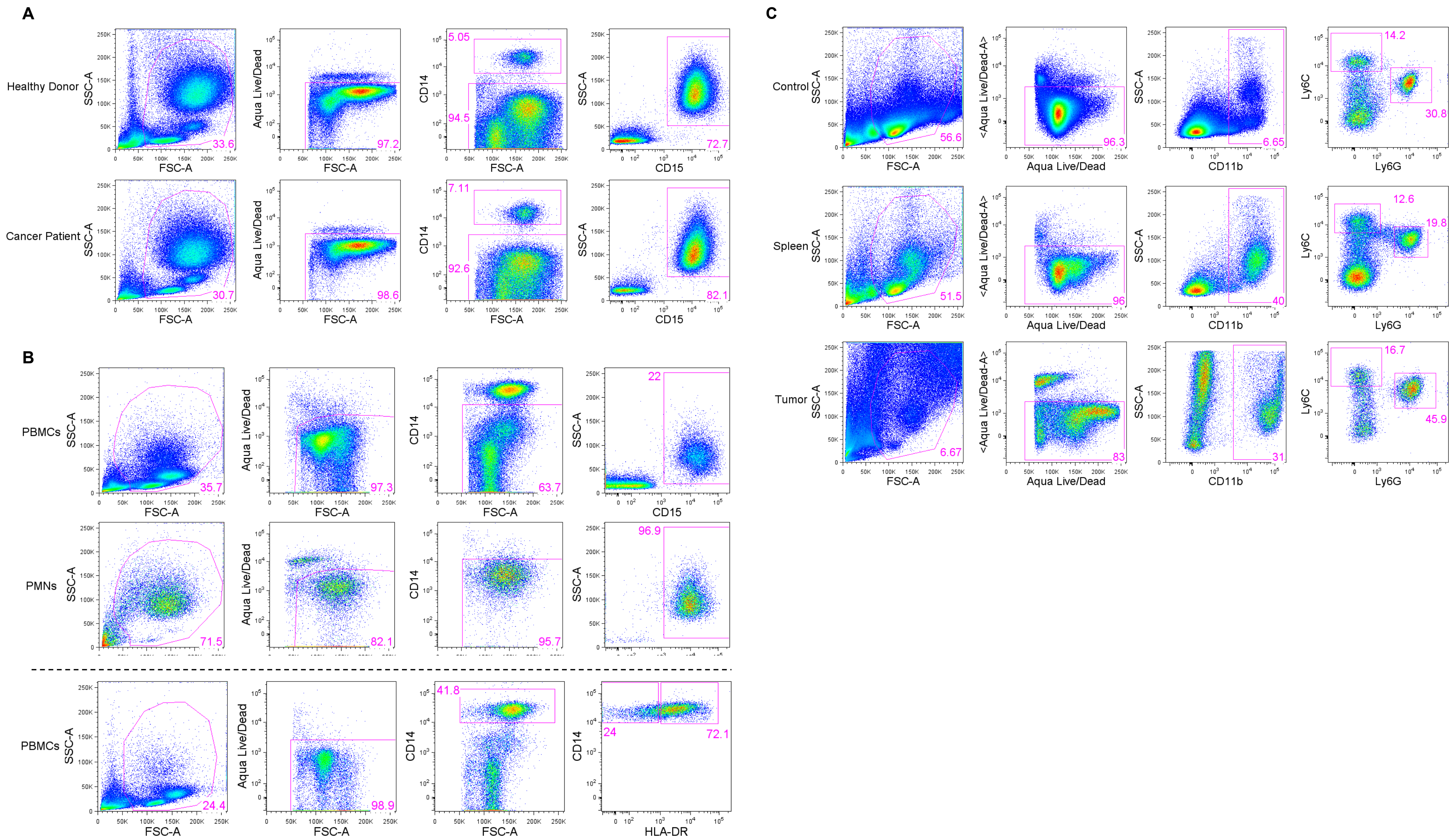

Supplementary Figure 2. Gating strategy for mouse and human MDSCs. (A) Gating strategy for CD15+ cells from whole blood of healthy donors and cancer patients. (B) Flow cytometry analysis of cancer patient's PMN-MDSCs versus PMNs based on CD15+ marker from PBMCs and PMNs fractions after histopaque gradient centrifugation (top). Flow cytometry gating for CD14+ M-MDSCs versus Mon based on HLA-DR expression (bottom), (C) phenotype of EL-4 tumor-bearing mice spleen and tumor PMN-MDSC (CD11b+Ly6CloLy6G+) and M-MDSC (CD11b +Ly6ChiLy6G-) compared with tumor-free mice by flow cytometry.

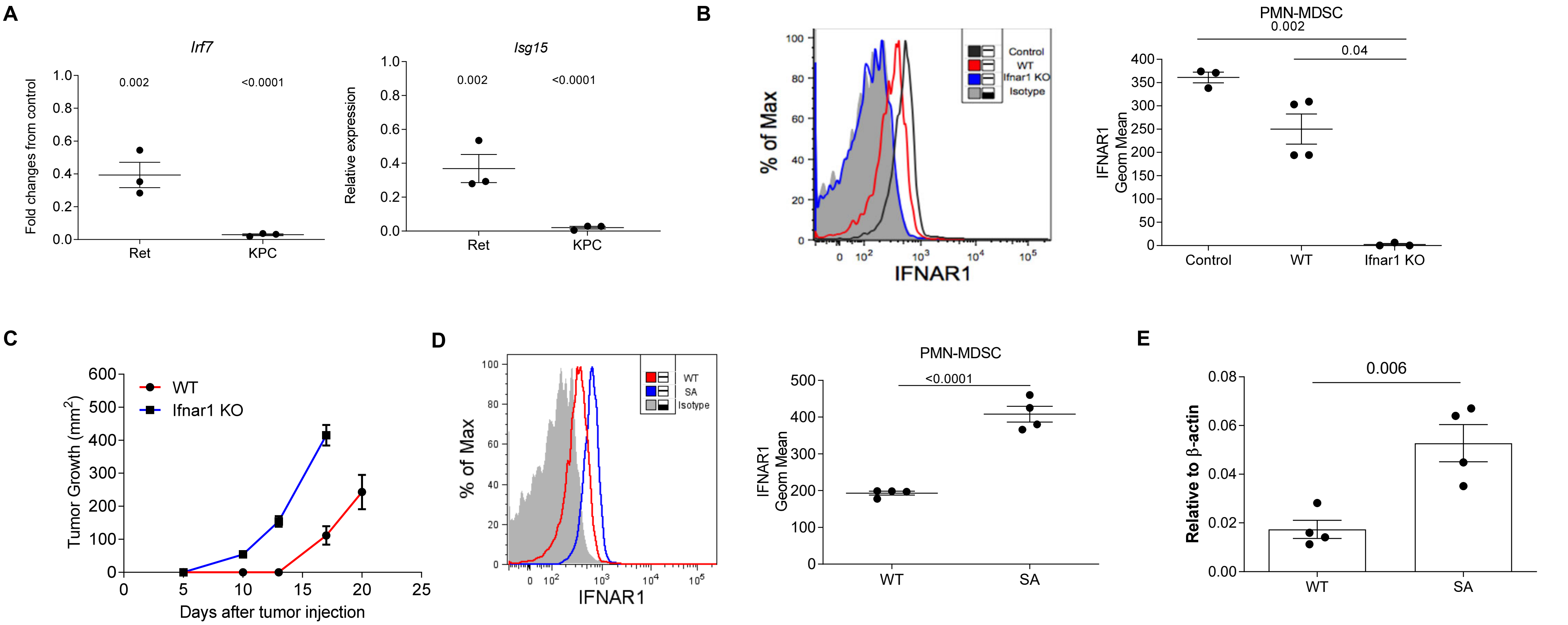

Supplementary Figure 3. Expression of IFN1 response genes in PMN-MDSC from KPC and RET transgenic mice and IFNAR1 levels in PMN-MDSCs from WT, IFNAR1 KO and SA tumor-bearing mice. (A). Irf7 and Isg15 expression in PMN-MDSCs isolated from spleen of Ret Melanoma and KPC mice tumor-bearing mice. Each dot represents gene expression as fold change over PMNs from spleen of tumor-free mice. (n=3). Mean ± SEM are shown. P values were calculated in unpaired two-sided Student's t-test. (B) Histogram (left) and cumulative results (right) of IFNAR1 expression in Ly6G+ cells (PMN-MDSCs) from spleen of control tumor-free or WT and IFNAR1 KO tumor-bearing mice. (n=4 for WT group and n=3 for other two groups). Mean ± SEM are shown. P values were calculated in one-way ANOVA test with correction for multiple comparisons. (C). EL-4 tumor growth in WT and Ifnar1 KO C57BL/6 mice. Representative of two experiments (n=3). Mean ± SEM are shown. (D) Histogram (left) and cumulative results (right) of IFNAR1 expression in Ly6G+ cells (PMN-MDSCs) from spleen of WT and SA tumor-bearing mice. Results of individual mice and mean ± SEM are shown. (n=4). P value was calculated in unpaired two-sided Student's t-test. (E) Expression of Irf7 in PMN-MDSC isolated from spleen of WT and SA EL4 TB mice. Results of individual experiments (n=4) and Mean ± SEM are shown. P value was calculated in unpaired two-sided Student's t-test.

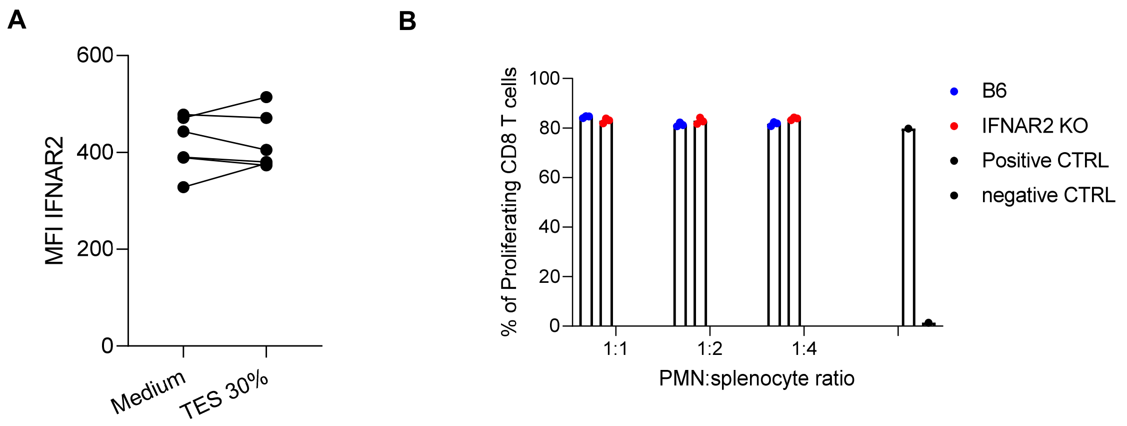

Supplementary Figure 4. Lack of IFNAR2 did not convert PMN to PMN-MDSC. A. PMN were isolated from blood of healthy donor volunteers and cultured for 16-18h with medium containing 20ng/ml ofGM-CSF with or without 30% of TES. IFNAR2 level was measured by flow cytometry. Each dot represent an individual volunteer. Geometric MFI is shown. B. PMN were isolated from BM of naïve WT or Ifnar2 KO mice and suppressive activity of the cells was measured by co-culturing PMN with PMEL splenocytes stained with Cell trace Far red at different ratio stimulated with the cognate peptide (gp100 10 ng/ml). Cell trace dilution was measured after 48h to assess proliferation of CD8 + T cells (n=3).

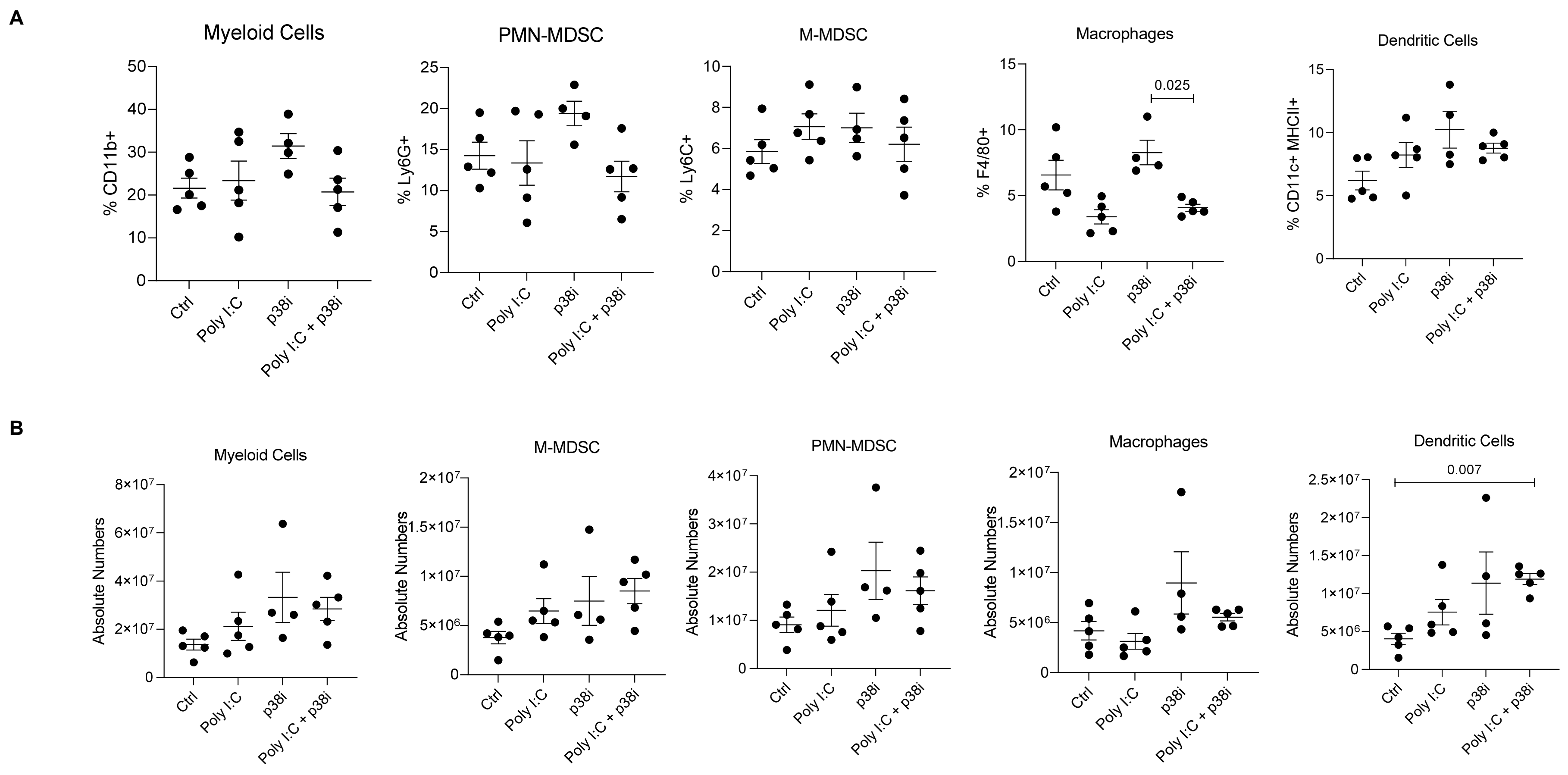

Supplementary Figure 5. Phenotype of myeloid cells after combination therapy. (A) Percentage of myeloid cells in spleen of MC38 tumor-bearing mice after control (ctrl), Poly I:C, p38i or Poly I:C + p38i (n=5). (B) Absolute numbers of myeloid cells in spleen of MC38 tumor-bearing mice after control
(ctrl), Poly I:C, p38i or Poly I:C + p38i (n=4 for p38i group and n=5 for all other groups). Mean ± SEM are shown. P values were calculated in one-way ANOVA test with correction for multiple comparisons.

Supplementary Table 1. Antibodies used in the study

Company	Antibody/isotype	Clone	Catalog number	Dilution used in the study
Biolegend	Anti-mouse IFNAR1	MARI-5A3	127312	1:50
	IgG1 κ Iso – PE	MOPC-21	400112	1:50
	CD11b – BV421	M1/70	101236	1:100
	CD11c – APC-Cy7	N418	117324	1:50
	Pe mouse IgG2a ISO	MOPC-173	400214	1:5
	Ly6G-FITC	1A8	127606	1:100
	Ly6C-APC	HK1.4	128016	1:200
	CD8a	53-6.7	100708	1:100
	Ly6C-PercP-Cy5.5	HK1.4	128012	1:200
PBL Assay Science	Hu-IFN-alpha/beta R2 PE	MMHAR-2	21385-3	1:5
BD Bioscience	Ly6C – PeCy7	AL-21	560593	1:100
	Ly6G – APC	1A8	560599	1:100
	I-A/I-E – PerCP-Cy5.5	M5/114.15.2	562363	1:100
	CD15 – PerCP-Cy5.5	HI98	560828	1:50
	CD14 – APC-H7	M5E2	561384	1:50
	HLA-DR – FITC	G46-6	560944	1:50
Thermo Fisher Scientific	F4/80 – FITC	BM8	11-4801-82	1:50
	p-p38 – APC	4NIT4KK	17-9078-42	1:10
	IgG2b κ Iso – APC	eBMG2b	17-4732-81	1:10
R&D system	Human IFN-alpha/beta R1 – PE	85228	FAB245P	1:10
	Mouse IgG1 – PE	11711	IC002P	1:10
	phosphoSer-IFNAR1 (western)	n/a	[1]	1:2000
	IFNAR1 (IP)	EA12	[2]	n/a
Abcam	IFNAR1 (western)	EP899Y	ab45172	1:1000
Sigma Aldrich	Ub (western)	FK2	04-263	1:1000
	Actin (western)	AC-15	A5441	1:1000
Millipore	anti-rabbit-HRP conjugated	n/a	AP187P	1:20000
Cell Signaling	anti-phospho-p38	28B10	9216S	1:1000
	anti-mouse-HRP conjugated	n/a	7076S	1:20000
Santa Cruz Biotech	anti-p38	C-20	sc-535	1:1000

Supplementary Table 2. Primers used in this study

Primer	Sequences
β-actin	F: GGA GGG GGT TGA GGT GTT
	R: GTG TGC ACT TTT ATT GGT CTC AA
Irf7	F: TGC TGT TTG GAG ACT GGC TA
	R: CGA AAT GCT TCC AGG GTA CG
Isg15	F: GCT CGAC TAA CTC CAT GAC GG
	R: CTC ACC AGG ATG CTC AGA GG
Arg1	R: ATC GGA GCG CCT TTC TCA AAA
	F: GGT CTC TCA CGT CAT ACT CTG TTT C
Ptgs2	F: GCC TGA GCG GGA ATA GTA GG
	R: GAT TTC CGG CTA GAG GTG GG
Ptges	F: GCA CAC TGC TGG TCA TCA AG
	R: ACG TTT CAG CGC ATC CTC
Nox2	R: GGG AAC TGG GCT GTG AAT GA
	F: CAG TGC TGA CCC AAG GAG TT

Supplementary References

1. Bhattacharya, S., et al., *Inducible priming phosphorylation promotes ligand-independent degradation of the IFNAR1 chain of Type I interferon receptor.* Journal of Biological Chemistry, 2010. **285**(4): p. 2318-2325.
2. Goldman, L.A., et al., *Characterization of Antihuman IFNAR-1 Monoclonal Antibodies: Epitope Localization and Functional Analysis.* Journal of Interferon & Cytokine Research, 1999. **19**(1): p. 15-26.

Supplementary Figure 6: Unprocessed gels

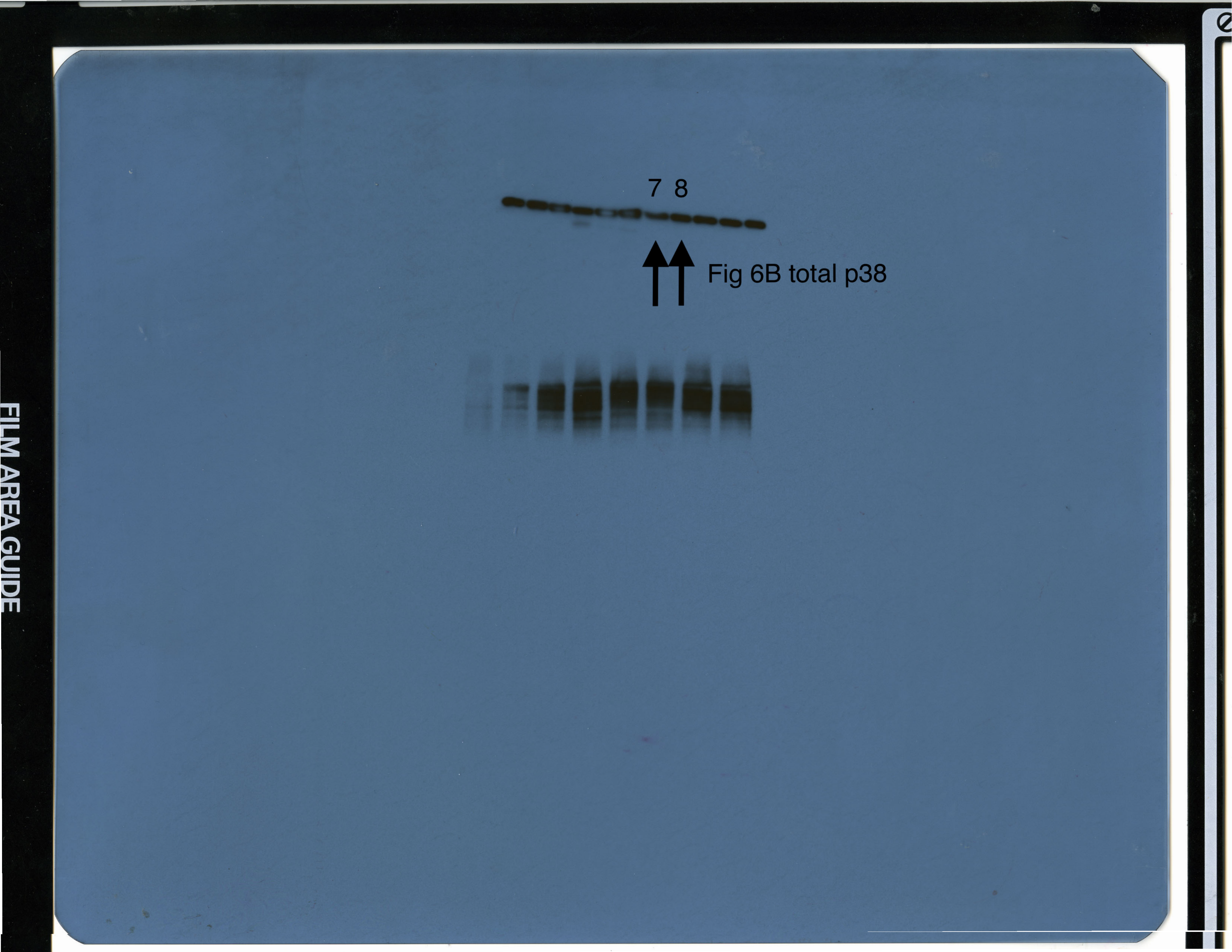

7 8
Fig 6B total p38
FILM AREA GUIDE

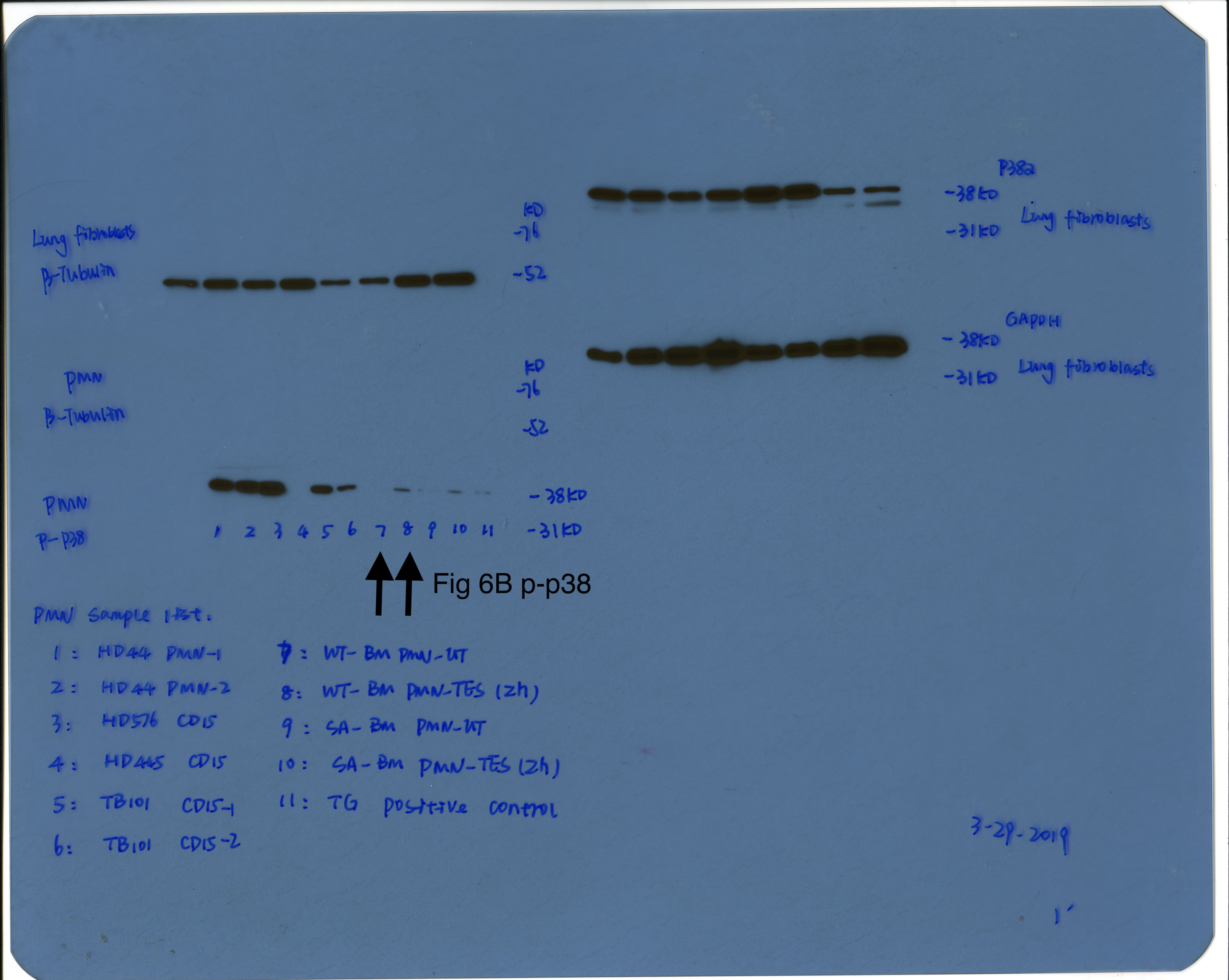

FILM AREA GUIDE

Fig 6F

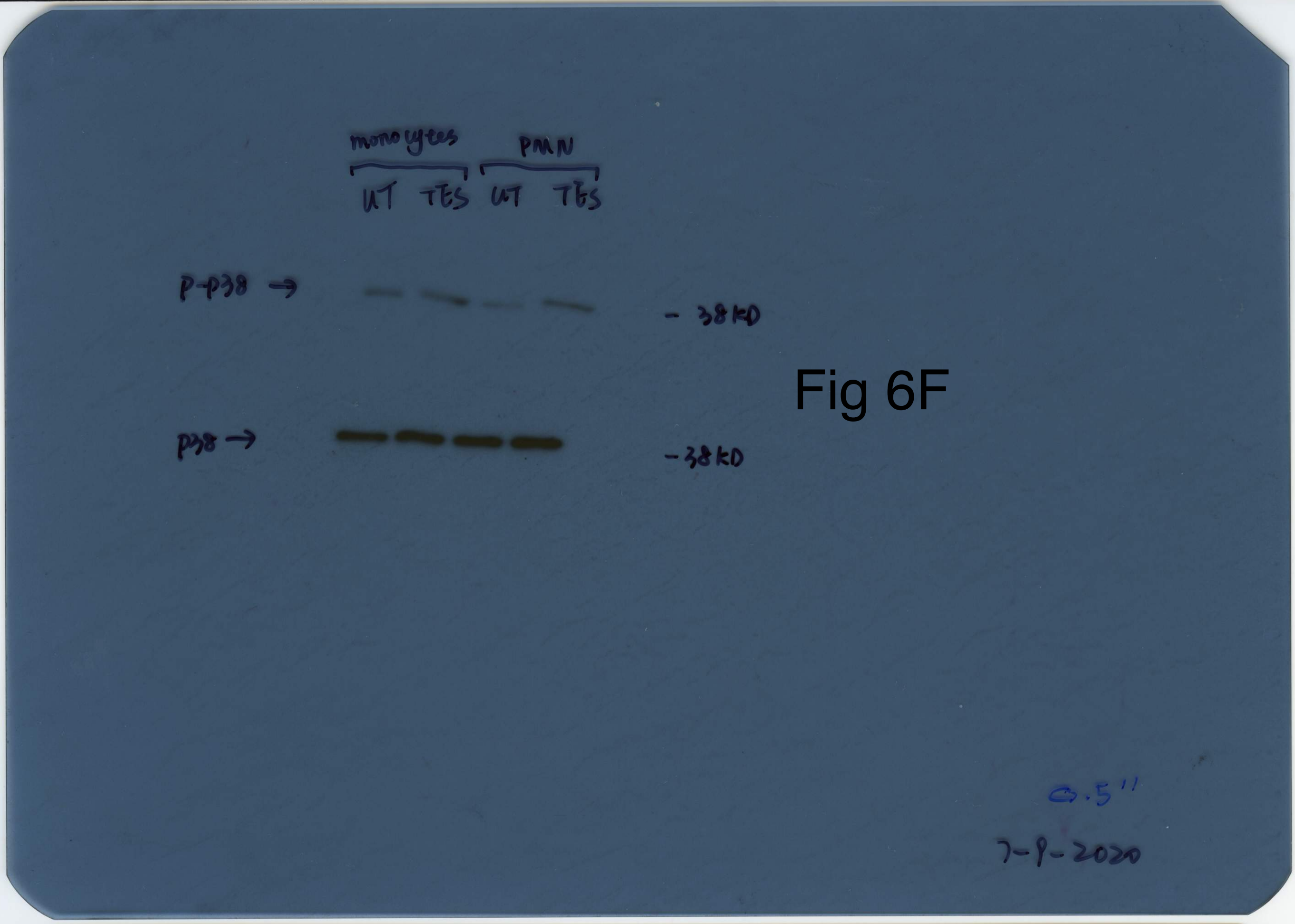
monocytes
PMN
UT TES UT TES
P-P38 →
- 38KD
P38 →
-38KD
0.5''
7-9-2020

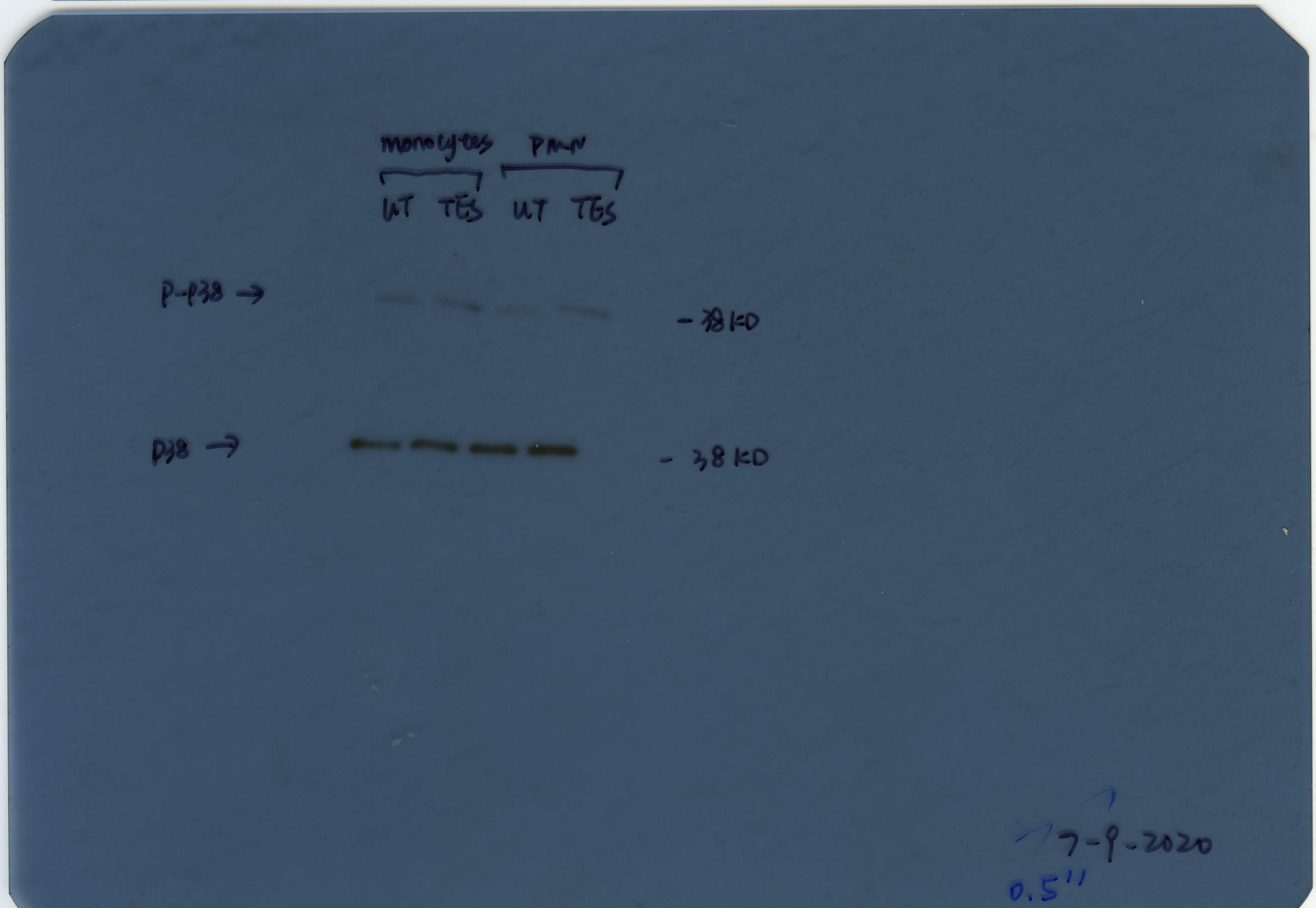
monocytes
PMN
UT TES UT TES
P-P38 →
- 38KD
P38 →
- 38KD
7-9-2020
0.5''

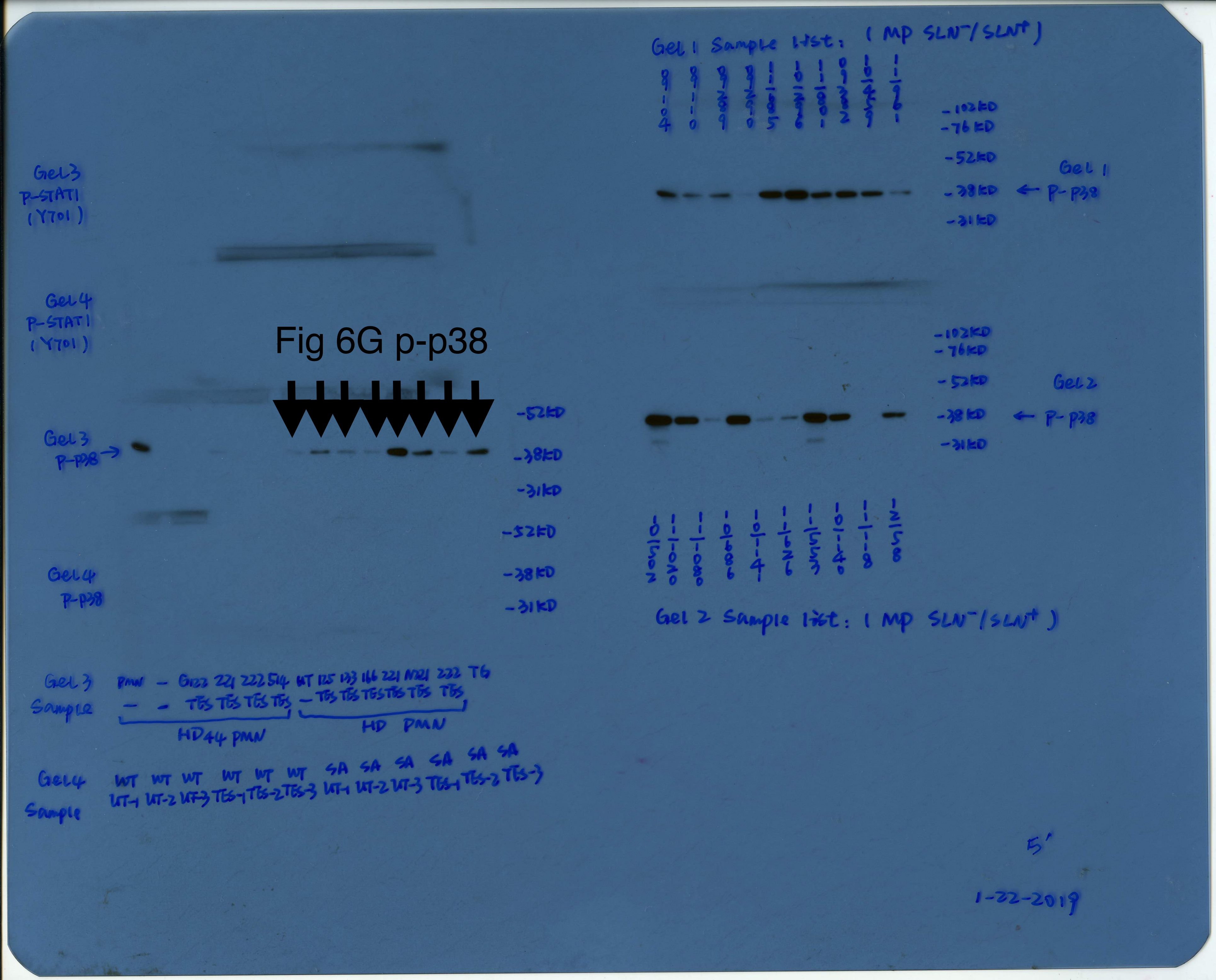

Gel 1 Sample list: (MP SLN-/SLN+)
-102KD
-76KD
-52KD
-38KD
-31KD
Gel 1
← P-p38
Gel 3
P-STAT1
(Y701)
Gel 4
P-STAT1
(Y701)
Fig 6G p-p38
Gel 3
P-p38 →
-52KD
-38KD
-31KD
Gel 4
P-p38
Gel 2
← P-p38
Gel 2 Sample list: (MP SLN-/SLN+)
Gel 3
Sample
PMN
HD44 PMN
HD PMN
Gel 4
Sample
WT UT-1 WT UT-2 WT UT-3 WT TES-1 WT TES-2 WT TES-3
SA UT-1 SA UT-2 SA UT-3 SA TES-1 SA TES-2 SA TES-3
5'
1-22-2019

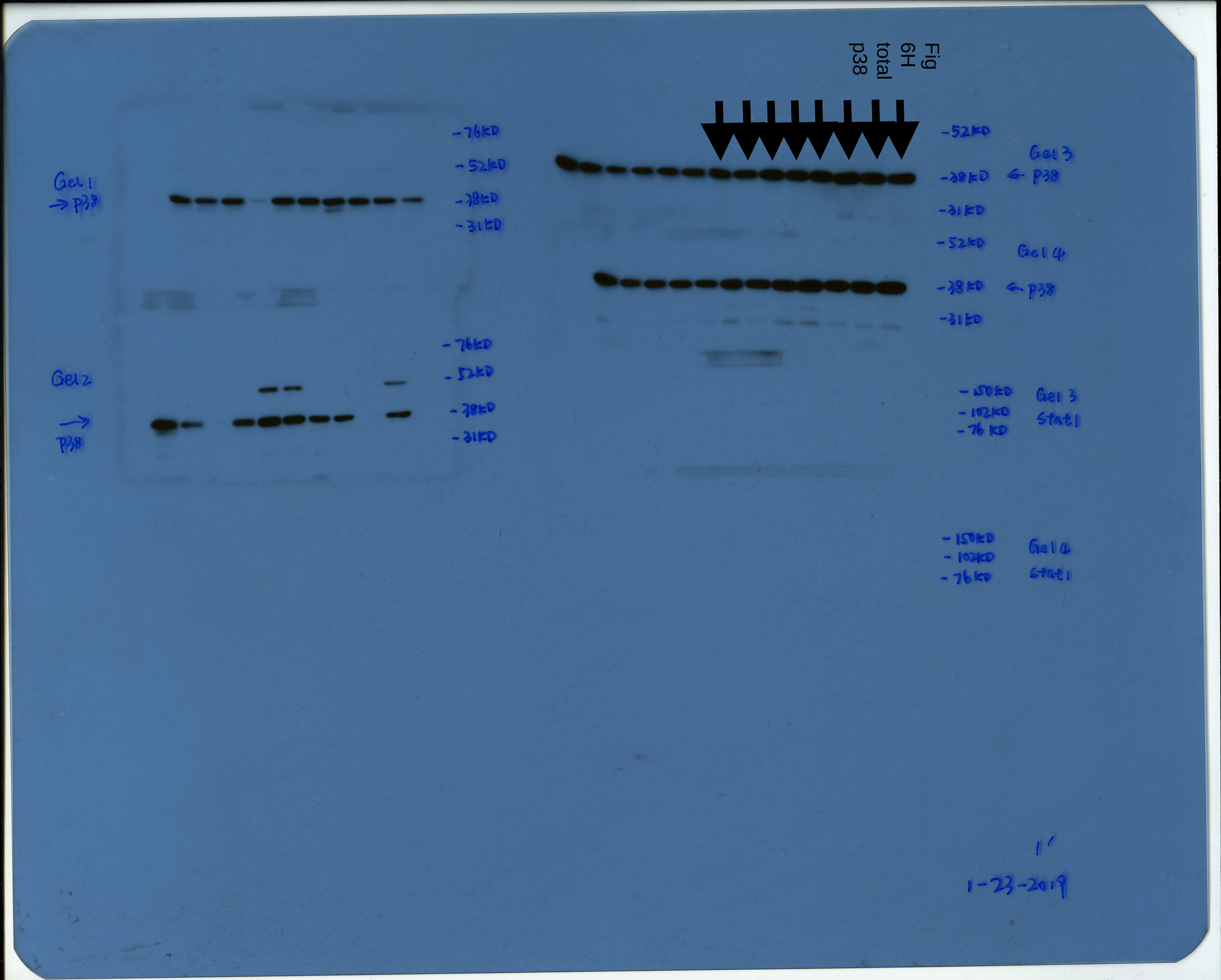
Fig 6H total p38
Gel 1
→ p38
-76KD
-52KD
-38KD
-31KD
Gel 2
→ p38
-76KD
-52KD
-38KD
-31KD
-52KD
-38KD ← p38
-31KD
Gel 3
-52KD
-38KD ← p38
-31KD
Gel 4
-150KD
-102KD
-76KD
Gel 3
Stat1
-150KD
-102KD
-76KD
Gel 4
Stat1
1'
1-23-2019

Western blots figure 6G

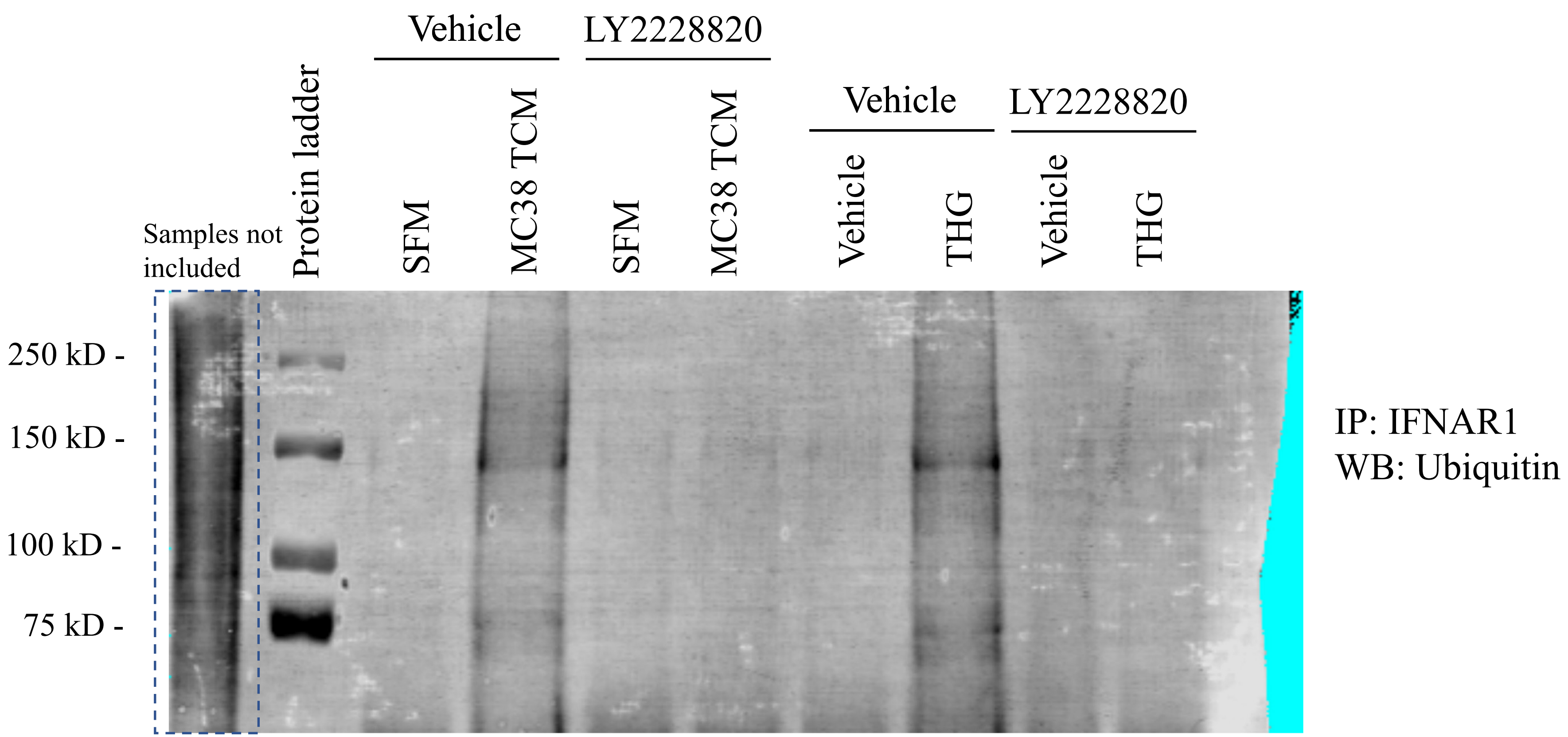

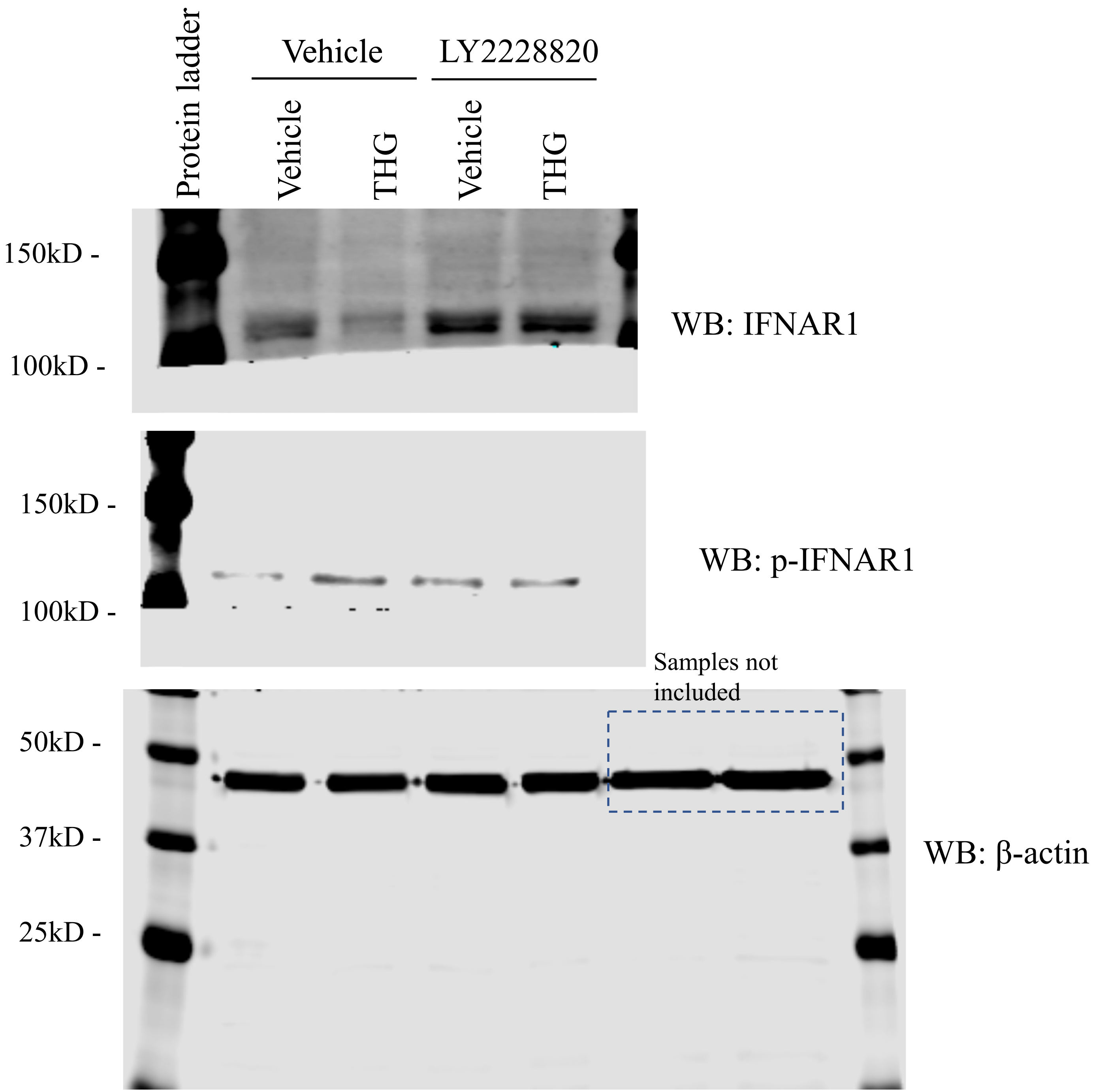
Protein ladder
Vehicle
LY2228820
Vehicle
THG
Vehicle
THG
150kD -
100kD -
WB: IFNAR1
150kD -
100kD -
WB: p-IFNAR1
Samples not included
50kD -
37kD -
25kD -
WB: β-actin

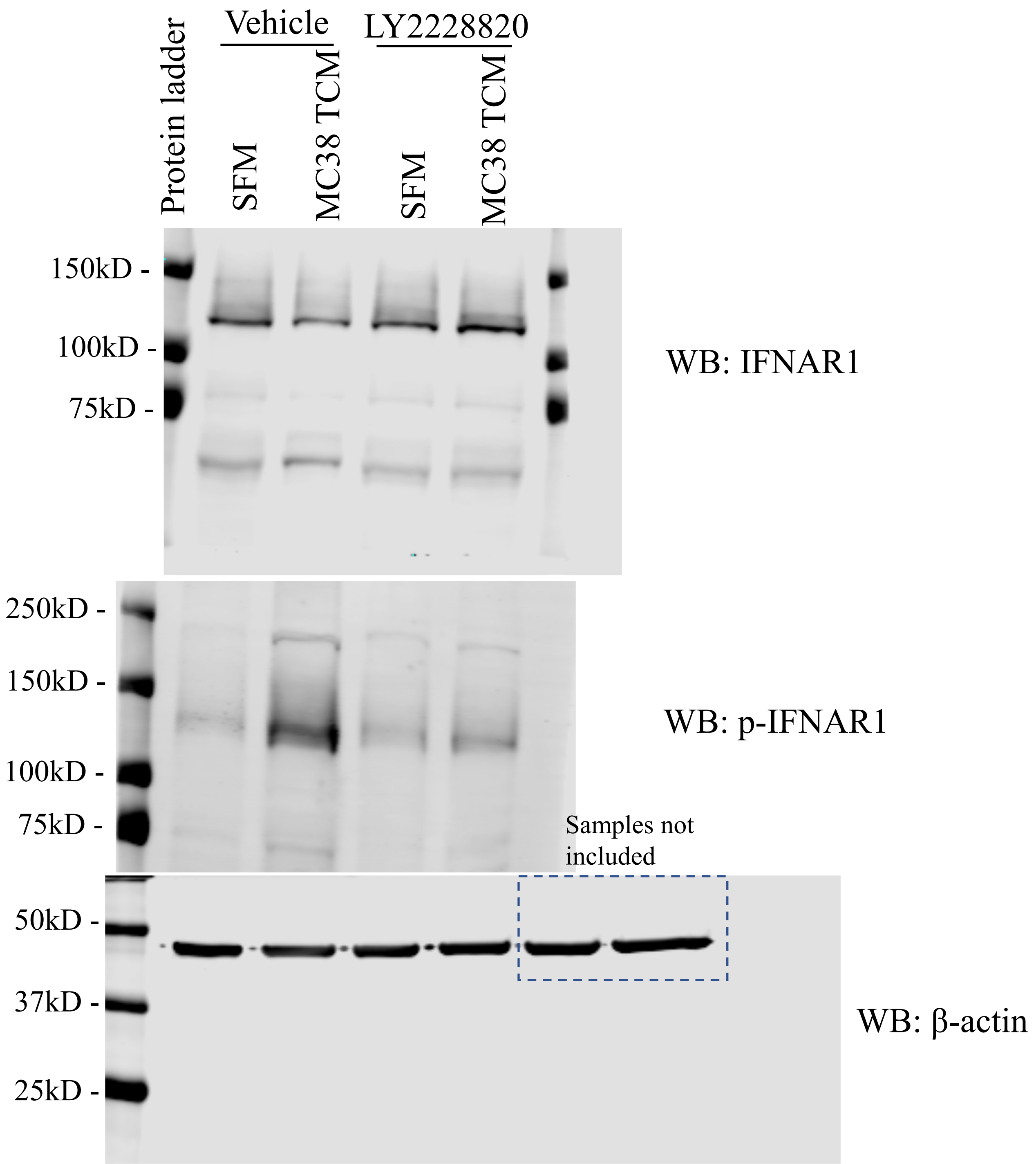
Protein ladder
Vehicle
LY2228820
SFM
MC38 TCM
SFM
MC38 TCM
150kD -
100kD -
75kD -
WB: IFNAR1
250kD -
150kD -
100kD -
75kD -
WB: p-IFNAR1
Samples not included
50kD -
37kD -
25kD -
WB: β-actin
