# Supplementary material for: Immune suppressive activity of myeloid-derived suppressor cells in cancer requires inactivation of the type I interferon pathway
Source: Nat Commun. 2021 Mar 19;12:1717. doi: 10.1038/s41467-021-22033-2 (PMC7979850; doi:10.1038/s41467-021-22033-2)
Supplement: Supplementary file 3 — Reporting Summary [file 41467_2021_22033_MOESM3_ESM.pdf]

nature research

Corresponding author(s): Dmitry Gabrilovich

Last updated by author(s): Feb 19, 2021

Reporting Summary

Nature Research wishes to improve the reproducibility of the work that we publish. This form provides structure for consistency and transparency in reporting. For further information on Nature Research policies, see our Editorial Policies and the Editorial Policy Checklist.

Statistics

For all statistical analyses, confirm that the following items are present in the figure legend, table legend, main text, or Methods section.

n/a	Confirmed	
☐	☒	The exact sample size (*n*) for each experimental group/condition, given as a discrete number and unit of measurement
☐	☒	A statement on whether measurements were taken from distinct samples or whether the same sample was measured repeatedly
☐	☒	The statistical test(s) used AND whether they are one- or two-sided<br>*Only common tests should be described solely by name; describe more complex techniques in the Methods section.*
☒	☐	A description of all covariates tested
☒	☐	A description of any assumptions or corrections, such as tests of normality and adjustment for multiple comparisons
☐	☒	A full description of the statistical parameters including central tendency (e.g. means) or other basic estimates (e.g. regression coefficient) AND variation (e.g. standard deviation) or associated estimates of uncertainty (e.g. confidence intervals)
☐	☒	For null hypothesis testing, the test statistic (e.g. *F*, *t*, *r*) with confidence intervals, effect sizes, degrees of freedom and *P* value noted<br>*Give P values as exact values whenever suitable.*
☒	☐	For Bayesian analysis, information on the choice of priors and Markov chain Monte Carlo settings
☒	☐	For hierarchical and complex designs, identification of the appropriate level for tests and full reporting of outcomes
☒	☐	Estimates of effect sizes (e.g. Cohen's *d*, Pearson's *r*), indicating how they were calculated

Our web collection on statistics for biologists contains articles on many of the points above.

Software and code

Policy information about availability of computer code

Data collection	No code was used
Data analysis	Bowtie2, RSEM v1.2.12 software, QIAGEN's Ingenuity® Pathway Analysis software , GraphPad Prizm 8.4.2

For manuscripts utilizing custom algorithms or software that are central to the research but not yet described in published literature, software must be made available to editors and reviewers. We strongly encourage code deposition in a community repository (e.g. GitHub). See the Nature Research guidelines for submitting code & software for further information.

Data

Policy information about availability of data

All manuscripts must include a data availability statement. This statement should provide the following information, where applicable:
- Accession codes, unique identifiers, or web links for publicly available datasets
- A list of figures that have associated raw data
- A description of any restrictions on data availability

RNAseq data were deposited to GEO with accession number GSE166770.

Field-specific reporting

Life sciences study design

All studies must disclose on these points even when the disclosure is negative.

Sample size	Sample size was determined using expected mean and standard deviation based on previous experience with similar type of experiments with type I error of 0.05 and power of more than 80%. Relevant citations are provided in manuscript
Data exclusions	No data were excluded from the analysis
Replication	At least three biological replicates were used in each experiment. All data were replicated. If results were not replicated they were reported as negative.
Randomization	In mouse treatment experiment simple randomization was performed after tumor inoculation before start of treatment by randomly assigning mice to the treatment group. By the time of randomization tumor size was not visible thus excluding bias In other experiments, cells from the same mice were split and were either left untreated or treated with indicated compounds to assess the biological effect. In this case the baseline was exactly the same.
Blinding	Specific blinding was not performed because of exploratory nature of the study. However, the person who perform measurements was not informed about the goals of the study and the nature of the treatment groups.

Reporting for specific materials, systems and methods

We require information from authors about some types of materials, experimental systems and methods used in many studies. Here, indicate whether each material, system or method listed is relevant to your study. If you are not sure if a list item applies to your research, read the appropriate section before selecting a response.

Materials & experimental systems

n/a	Involved in the study	
	x	Antibodies
		Eukaryotic cell lines
x		Palaeontology and archaeology
	x	Animals and other organisms
	x	Human research participants
x		Clinical data
x		Dual use research of concern

Methods

n/a	Involved in the study	
x		ChIP-seq
	x	Flow cytometry
x		MRI-based neuroimaging

Antibodies

Antibodies used

All antibodies used in the study are reported in supplemental table 1.
Biolegend Anti-mouse IFNAR1 MARI-5A3 127312 1:50

IgG1 κ Iso – PE MOPC-21 400112 1:50

CD11b – BV421 M1/70 101236 1:100

CD11c – APC-Cy7 N418 117324 1:50

Pe mouse IgG2a ISO MOPC-173 400214 1:5

Ly6G-FITC 1A8 127606 1:100

Ly6C-APC HK1.4 128016 1:200

CD8a 53-6.7 100708 1:100

Ly6C-PercP-Cy5.5 HK1.4 128012 1:200

PBL Assay Science Hu-IFN-alpha/beta R2 PE MMHAR-2 21385-3 1:5

BD Bioscience Ly6C – PeCy7 AL-21 560593 1:100

Ly6G – APC 1A8 560599 1:100

I-A/I-E – PerCP-Cy5.5 M5/114.15.2 562363 1:100

	CD15 – PerCP-Cy5.5 HI98 560828 1:50<br><br>CD14 – APC-H7 M5E2 561384 1:50<br><br>HLA-DR – FITC G46-6 560944 1:50<br><br>Thermo Fisher Scientific F4/80 – FITC BM8 11-4801-82 1:50<br><br>p-p38 – APC 4NIT4KK 17-9078-42 1:10<br><br>IgG2b κ Iso – APC eBMG2b 17-4732-81 1:10<br><br>R&D system Human IFN-alpha/beta R1 – PE 85228 FAB245P 1:10<br><br>Mouse IgG1 – PE 11711 IC002P 1:10<br><br>p38 (western)<br><br>p-p38 (western)<br><br>IFNAR1 (IP) EA12 n/a<br><br>Abcam IFNAR1 (western)<br><br>Sigma Aldrich Ub (western) FK2<br><br>Actin (western) AC-15<br><br>anti-rabbit-HRP conjugaed<br><br>Cell Signaling anti-phospho-p38<br><br>Santa Cruz Biotech anti-p38
Validation	All antibodies were obtained from commercial sources with reported validation.

Eukaryotic cell lines

Policy information about cell lines

Cell line source(s)	EL4 lymphoma, LLC (Lewis lung carcinoma), CT-26 colon carcinoma, B16F10 melanoma, PCI-30 (human head and neck squamous carcinoma) were obtained from ATCC. MC38 colon carcinoma from Dr. Turkova, University of Pittsburgh), MethA sarcoma was obtained from Dr. Lloyd J. Old.
Authentication	Cell lines were authenticated by ATCC. Information about the details of authentication may be obtained from ATCC.
Mycoplasma contamination	All cell lines were tested for mycoplasma every 6 months and found to be negative
Commonly misidentified lines<br>(See ICLAC register)	We did not use commonly misidentified lines.

Animals and other organisms

Policy information about studies involving animals; ARRIVE guidelines recommended for reporting animal research

Laboratory animals	We used mice, C57BL/6 strain 6-8 weeks old of both sexes.
Wild animals	No wild animals were used
Field-collected samples	The study did not involve field-collected samples
Ethics oversight	Study was approved by IACUC of Wistar Institute

Note that full information on the approval of the study protocol must also be provided in the manuscript.

Human research participants

Policy information about studies involving human research participants

Population characteristics	PB was collected from i) 12 patients with non-small cell lung cancer and 5 with small cell lung cancer; (ii) 5 patients with breast cancer, (iii) 10 patients with colorectal cancer, (iv) 4 patients with pancreatic adenocarcinomas, (v) 3 patients with esophageal cancer; (vi) 3 patients with head and neck cancer, (vii) 1 patient with gastric cancer, and (viii) 1 patient had renal cancer. In some patients with lung, colorectal, renal or head and neck cancer, tumor tissues (0.2 - 1g) were surgically removed. Ages of cancer patients were between 46 and 92 years (median, 70 years), 24 males and 20 females. Peripheral sample of blood from 21 healthy donors with ages 30 to 64 (median, 55 years), 7 males 14 females and were used as control for cancer patients or for in vitro experiments.
Recruitment	Participants were recruited by participating physiciaas. Patients were not selected for treatment and samples of blood or tumor tissues were collected during standard clinical procedures. Results of the study for individual patients were not available for participating physicians or patient, which exclude bias.
Ethics oversight	The study was approved by the institutional review boards (IRBs) of the Christiana Care Health System at Helen F. Graham Cancer Center and The Wistar Institute. All the patients and healthy donors signed IRB approved consent forms.

Note that full information on the approval of the study protocol must also be provided in the manuscript.

Flow Cytometry

Plots

Confirm that:

- [x] The axis labels state the marker and fluorochrome used (e.g. CD4-FITC).
- [x] The axis scales are clearly visible. Include numbers along axes only for bottom left plot of group (a 'group' is an analysis of identical markers).
- [x] All plots are contour plots with outliers or pseudocolor plots.
- [x] A numerical value for number of cells or percentage (with statistics) is provided.

Methodology

Sample preparation	Single-cell suspensions of BM, spleen and tumors were prepared, and red cells were removed using ACK lysing buffer. In other experiments, cells were culture in vitro before flow cytometry analysis was performed. All antibodies incubations were performed for 15 min at 4"C in dark and all centrifugation was done at 1,500 r.p.m. at 4"C for 5 min. Usually up to lx106 cells were incubated with Fe-block (BD Biosciences) for 10 min and surface staining was performed at 4°C for 15 min.
Instrument	Cells were run on LSRII flow cytometer (BD Biosciences)
Software	Data were analyzed by FlowJo (Tristar).
Cell population abundance	Purity of the populations after sort was more than 98%.
Gating strategy	Gating strategy including live/dead discrimination, elimination of duplexes using FSC/SSC, gating on size and granularity to narrow specific populations (FSC/SSC) and gating on specific markers. Damp channels and isotype controls were used to set-up the gates.

- [x] Tick this box to confirm that a figure exemplifying the gating strategy is provided in the Supplementary Information.
